# Bone Marrow Adipose Tissue: To Be or Not To Be a Typical Adipose Tissue?

**DOI:** 10.3389/fendo.2016.00085

**Published:** 2016-06-30

**Authors:** Pierre Hardouin, Tareck Rharass, Stéphanie Lucas

**Affiliations:** ^1^Laboratory of Pathophysiology of Inflammatory Bone Diseases PMOI, University of Littoral-Opale Coast ULCO, Boulogne sur Mer, France

**Keywords:** fat–bone association, marrow fat, adipokine, bone marrow adiposity, skeletal adipocyte, osteoporosis, bone fragility

## Abstract

Bone marrow adipose tissue (BMAT) emerges as a distinct fat depot whose importance has been proved in the bone–fat interaction. Indeed, it is well recognized that adipokines and free fatty acids released by adipocytes can directly or indirectly interfere with cells of bone remodeling or hematopoiesis. In pathological states, such as osteoporosis, each of adipose tissues – subcutaneous white adipose tissue (WAT), visceral WAT, brown adipose tissue (BAT), and BMAT – is differently associated with bone mineral density (BMD) variations. However, compared with the other fat depots, BMAT displays striking features that makes it a substantial actor in bone alterations. BMAT quantity is well associated with BMD loss in aging, menopause, and other metabolic conditions, such as anorexia nervosa. Consequently, BMAT is sensed as a relevant marker of a compromised bone integrity. However, analyses of BMAT development in metabolic diseases (obesity and diabetes) are scarce and should be, thus, more systematically addressed to better apprehend the bone modifications in that pathophysiological contexts. Moreover, bone marrow (BM) adipogenesis occurs throughout the whole life at different rates. Following an ordered spatiotemporal expansion, BMAT has turned to be a heterogeneous fat depot whose adipocytes diverge in their phenotype and their response to stimuli according to their location in bone and BM. *In vitro, in vivo*, and clinical studies point to a detrimental role of BM adipocytes (BMAs) throughout the release of paracrine factors that modulate osteoblast and/or osteoclast formation and function. However, the anatomical dissemination and the difficulties to access BMAs still hamper our understanding of the relative contribution of BMAT secretions compared with those of peripheral adipose tissues. A further characterization of the phenotype and the functional regulation of BMAs are ever more required. Based on currently available data and comparison with other fat tissues, this review addresses the originality of the BMAT with regard to its development, anatomy, metabolic properties, and response to physiological cues.

Besides being the main energy storage sites, adipose tissues have revealed through these last decades their diversity regarding their cellular composition, anatomical location, and pathophysiological properties. Indeed, adipocytes are typically classified into three categories: white, brown, and beige types (1). White adipocytes store excessive energy supply in a unilocular triglyceride droplet to release fatty acids in periods of energy depletion. White adipocytes also exert an endocrine function through the secretions of various adipokines that mainly regulate metabolism and inflammation (2). Conversely, brown adipocytes are multilocular, rich in mitochondria, and dissipate the energy into heat through the uncoupling protein-1 (UCP-1). Their high glucose uptake and oxidative capacities make them key players in the energy balance. At last, beige (also called “brite”) adipocytes are brown-like adipocytes with UCP-1 expression that arise within white fat depots in response to cold or catecholaminergic stimulation (3).

Most adipose tissues in adult humans consist of white adipose tissue (WAT) that encompasses major subcutaneous depots (85% of total adipose tissue) in the lower or abdominal body parts and visceral fat depots (~10%) with omental, mesenteric, or retroperitoneal distributions. According to that anatomical location and the pathophysiological contexts, WATs exhibit differences in their development pattern, lipogenic and lipolytic activities, “browning” ability, or endocrine functions. This is best exemplified by the development of visceral adipose tissue that is considered as a strong predictive factor for the emergence of obesity co-morbidities, notably through the secretion of pro-inflammatory cytokines and an enhanced lipolysis (4). Brown adipose tissue (BAT) exhibits a more diffuse distribution with discrete small depots. Owing to the recent reassessement of BAT in adults, stimulating BAT and beige adipocyte recruitment have become promising strategies for the management of metabolic diseases (3, 5).

In addition to WAT and BAT, bone marrow adipose tissue (BMAT) emerges as a “new” fat depot that could represent up to 5% of total fat mass in adults. The presence of BMAT – also referred to as “yellow” bone marrow (BM) – was of a long-standing knowledge but has been surprisingly disregarded for many decades. Meanwhile, bone has been revealed as a target and a regulator of energy metabolism: the two main adipokines, adiponectin and leptin, modulate bone mass through indirect and direct mechanisms (6, 7) and undercarboxylated osteocalcin, the bone-derived hormone, positively impacts on whole-body glucose metabolism (8, 9). Moreover, numerous clinical studies indicate strong relationships between BMAT amount and bone loss emphasizing its potential pathophysiological role in osteoporosis. Other lines of evidence support the involvement of BMAT in hematopoiesis regulation (10) and in the pathophysiology of myeloma (11) and bone metastases (12). Such bone–fat connections have contributed to renew interest in BMAT. Yet, the anatomical dissemination of BM fat and the difficulties to study adipocytes inside bones have considerably hampered our understanding of BM adipocyte (BMA) function and its relative contribution to pathophysiological processes compared with extramedullary fat depots. In this review, we aim at highlighting the current knowledge of BMAT development and phenotype to pinpoint its original features as an adipose tissue.

## BMAT Development is Both Physiological and Pathological

### Exploration of BMAT Development

The first descriptions of “hematopoietic red BM” replacement by the “fat yellow BM” were brought by histomorphometric studies of iliac crest biopsies in humans (13, 14) or other bone sites in animals. BMAT quantification has been, thus, performed using specific but static parameters (adipocyte number and diameter, percent of adipocyte volume per tissue volume), which precludes a reliable and dynamic assessment of adipocyte evolution according to the pathophysiological conditions. Magnetic resonance imaging (MRI) has been being of considerable interest to map non-invasively the distribution of hematopoietic BM and fatty BM in clinical studies (15). Moreover, proton magnetic resonance spectroscopy (1H MRS) allows the relative assessment of the saturated and unsaturated fatty acid composition of the fat fraction to monitor the lipid content changes. Combined with bone mineral density (BMD) and bone structure measurement by dual energy X-ray absorptiometry (DEXA) and high-resolution peripheral quantitative computed tomography, respectively, MR techniques have been being instrumental to follow BMAT development and to evaluate relationship between bone quantity and BMAT amount in diverse cohorts. Finally, using the lipid affinity of the opaque agent osmium tetroxide, a three-dimensional quantification of BMAT (whole amount, individual volume, spatial distribution) can be achieved by micro- or nanocomputerized tomography (μCT, nanoCT) in animal decalcified bones (16).

### Physiological BM Cellular Conversion to BMAT

Bone marrow adiposity development has been shown to be age, bone site, and gender dependent. At birth, bone cavities mainly contain active hematopoietic red marrow. BMAT accretion then occurs in an orderly and centripetal way: the process begins in the terminal phalanges around birth, continues in the appendicular skeleton (from the diaphysis to the distal and proximal extremities of the long bones) and finally arises in the axial skeleton (15, 17, 18). By the age of 25 years, BMAT is considered to occupy 50 (17) to 70% (16) of the BM volume, while hematopoietic BM is mainly restricted to the axial skeleton, ribs, sternum, and proximal metaphyses of humerus and femur. Afterward, the BM conversion into BMAT slowly progresses throughout the adulthood. Interestingly, women exhibit less BMAT amount compared with age-matched men (19, 20) prior the menopause age, while the following period is associated with a sharp increase of BM adiposity (21).

The development pattern in rodents is considered to be similar though it is far to be as well characterized as in humans. Histological studies of femur or tibia show the presence of BMA at adult age (22, 23), which further increases with aging (24, 25). Of note, the percentage of BM adiposity (or BMA density) appears low in rodents when compared with humans and varies according to the mouse strain. Moreover the presence of different BMA subsets could be suspected from early (26) to more recent studies (27, 28). However, it is with the new introduction of osmium tetroxide staining combined with μCT visualization that analysis of BMAT development has led to the first characterization of two BMA subpopulations in rodents (29). Scheller and collaborators propose to distinguish constitutive BMA (cBMA) – which arise first and early in life in distal tibia and caudal vertebrae to constitute a rather dense fat depot – from regulated BMA (rBMA) whose formation is late, increases with age, and occurs in a more scattered way in the proximal tibia, distal femur, and lumbar vertebrae (29). Importantly, this distinction based on a spatiotemporal distribution also corresponds to a different metabolic pattern and to different bone remodeling regions (see “Specific properties of BMA versus other adipocytes”). How such classification can be extrapolated to humans is still difficult although one can suggest that cBMA reside in the feet and hands, whereas the rBMA develops in the proximal femur and lumbar vertebrae (16, 29).

### BMAT Development Differs from that of Extramedullary Adipose Tissues

Formed during gestation, BAT quantity and activity are maximal at birth to provide an efficient thermogenesis during the first weeks. Beyond puberty that is characterized by an important BAT activity, cold-activated BAT incidence remains high in young adults but rapidly declines by the age of 30 to almost disappear in the elderly (30). BAT function and WAT browning also decrease with age in rodents (31) as shown in Table 1. Thus, the development pattern of BMAT differs from the recruitment of thermogenic adipose tissues during the lifespan and other physiological conditions [exercice, cold exposure (3, 5)], supporting that BMAT development responds to different cues.

**Table 1:** **Comparison of the main characteristics of brown adipose tissue (BAT), white adipose tissues (WAT), and bone marrow adipose tissue (BMAT) in rodents**.

	BAT	WAT	BMAT
Main locations	Interscapular (32)	Subcutaneous (inguinal) and visceral (perigonadal > mesenteric > retro-peritoneal) (32)	Constitutive BMA (cBMA): distal tibia and caudal vertebrae
			Regulated BMA (rBMA): proximal tibia and long bones, lumbar vertebrae (29)
Mean adipocyte diameter	–	For rat^a^	For rat^a^
		~56 μm for inguinal	~40 μm for caudal cBMA
		~74 μm for perigonadal	~33 μm for tibia rBMA
Amount variation during			
Aging	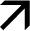 with “whitening” 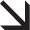 activity (33)	Subcutaneous 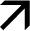 Visceral 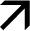 (33)	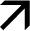 (mainly rBMA) (25, 29)
Calorie restriction (30%)	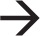 (34, 35)	Subcutaneous 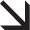 Visceral 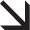 (34, 35) or unchanged (22, 35)	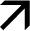 (mainly rBMA) (22, 34, 35)
Cold exposure	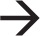 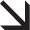 activity (36, 37)	Subcutaneous 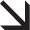 with beiging 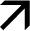 (36, 37)	rBMA 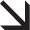 cBMA 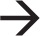 (29)
High-fat diet-induced obesity	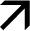 with “whitening” 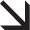 activity (38)	Subcutaneous 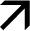 Visceral 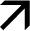 (39, 40)	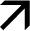 (reported for rBMA in long bones) (39–41)
Ovariectomy	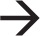 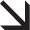 activity (42)	Subcutaneous 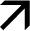 Visceral 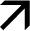 (42)	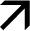 (reported for rBMA in long bones) (27)

*^a^Values from Sprague Dawley female rats aged between 19 and 24 weeks (29, 43)*.

*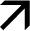 refers to “increased,” 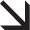 refers to “decreased,” 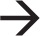 refers to “unchanged.” “Whitening” refers to BAT with a changed morphology as occurrence of unilocular adipocytes with increased triglyceride storage. “Beiging” refers to WAT with a changed morphology as the occurrence of multilocular adipocytes with UCP-1 expression*.

Early WAT formation is achieved through two early periods of intense precursor proliferation with adipocyte differentiation and lipogenesis in the meanwhile. By the end of adolescence, an adipocyte number set point specific to each individual is considered to be reached and to remain constant throughout life (44), with a rather low adipocyte renewal rate estimated at 8% per year (45). Indeed, the tremendous expandability of WAT primarily relies on adipocyte hypertrophy (cell size increase). Hyperplasia (cell number increase) occurs secondly when adipocyte storage capacity is exceeded and preferentially in subcutaneous AT. WAT growth is, thus, observed throughout the adult lifespan with a maximal mass achieved at middle–early old age. In advanced old age, white fat depots are redistributed with a subcutaneous fat loss in favor of visceral fat accumulation and ectopic fat deposition in other tissues (46). BMAT development with its long-lasting hyperplasia and concomitant hypertrophy (14) throughout life exhibits some striking differences compared with WAT. Moreover, BMAT amount is generally not correlated to usual anthropometric parameters of adiposity, such as waist-to-hip ratio, amount of visceral or subcutaneous fat, or even body mass index (47–49). Altogether, BMAT formation also appears independently regulated from extramedullary WAT both in humans and rodents (Table 1).

### Adipose Tissues and Relationship with Skeletal Fragility: The Importance of BMAT Development

Beyond being a simple witness of age, diffuse or local BMAT accumulation has been described in several types of osteoporosis notably that associated with aging, menopause, anorexia nervosa, or glucocorticoid treatment. In humans, BMAT amount is even found inversely correlated with bone quantity in aged (47, 50), post-menopausal (51), and anorexia nervosa (52) subjects at various bone sites. Enhanced BMAT formation is also depicted in animal models of aging (53), ovariectomy (27), calorie restriction (22), or following glucocorticoid administration (54). Bone loss results from an altered bone remodeling with either decreased number and/or mineralizing function of osteoblasts as in senile osteoporosis, or with increased bone resorption by osteoclasts that overwhelms bone formation as in post-menopausal osteoporosis (55). BMAT is suspected to contribute to this unbalanced bone remodeling through the adipogenesis process *per se* or its paracrine activity (see Section “BM Adipogenesis” and “Specific Properties of BMA”). However, it has to be emphasized that blocking BMAT formation failed to generate any bone modification in some mouse models (56, 57).

The impact of other adipose tissues on bone has of course been the subject of intensive clinical research that led to a complex picture. Whereas inconsistent results are drawn with subcutaneous adipose tissue measurements, visceral adipose tissue level, when directly quantitated, is often found negatively associated with BMD and bone quality (58). Indeed, excess visceral fat as in obesity is paralleled by an altered adipokine secretion with increased pro-inflammatory cytokines that have been suggested to interfere with bone remodeling (59). A positive association between BAT volume and BMD has been reported in a few studies in humans (60–62). Yet the prevalence and activity of BAT in humans remain difficult to measure. The beneficial impact of active BAT on bone has also been reported in mouse models (63, 64) and could rely on direct [derived-BAT adipokines (64)] or indirect mechanisms (61, 63). Moreover, bone fragility is a comorbidity of several metabolic diseases and BMAT evolution is obviously of interest in that context.

### BMAT Development in Metabolic Diseases

Regarding with type 1 diabetes, genetically or streptozotocin-induced insulin deficiency results in increased BMAT in the long bones of mice (65). However, preventing BMAT formation does not impact on the bone loss (66) inherent to these models and the disease. Moreover, as reported in one study, BMAT content measured at different bone sites was unchanged in diabetic patients compared with control subjects (67). Thus, the involvement of BMAT in type 1 diabetes remains unclear and deserves further explorations.

The skeletal health in obesity has been a controversial subject (68, 69). However, most clinical and epidemiologic studies have reported an alteration of bone quality in obesity leading to an increased fracture incidence at specific bone sites (59, 70–72). Even though visceral adipose tissue quantity can be negatively associated with bone microarchitecture and strength (73), BMAT content has been poorly examined in obesity. So far, only one clinical study performed in obese premenopausal women has reported a positive correlation between visceral adiposity and vertebral BMAT (74). The Ob/Ob mouse model with extreme obesity due to spontaneous leptin disruption shows increased BM adipogenesis in the long bones (75). Nevertheless, this model remains complex to analyze because of the pleiotropic effects of leptin on bone and metabolism. The BMA amount in the long bones also increases in models of high-fat diet-induced obesity (39, 41, 76) (Table 1). However, divergent results make it difficult to conclude about the bone phenotype in these models and no associations have been drawn between metabolic parameters and the BMAT rise yet. Of note, animal age and diet duration appear to influence BMAT increment (41, 76), so that older mice would develop more quickly and at a higher level BMA.

Type 2 diabetic patients exhibit an increased fracture risk that is predominantly linked to a compromised bone quality (77, 78). In post-menopausal women, BMAT content in vertebrae is found unchanged by the diabetic state (79, 80), even though it is higher in patients who experienced prolonged hyperglycemia reflected by a HbA1c level above 7% (79).

Weight loss is well known to trigger bone loss, as exemplified in anorexia nervosa but also in bariatric surgery in obesity care (59). In anorexia nervosa (52), BMAT unexpectedly develops while subcutaneous and essentially visceral adipose tissues are extremely depleted. Bariatric surgery (81) or a 4-week-calorie restriction (82) in obese women leads to a marked or a more modest reduction of extramedullary fat depots, respectively. Yet, these decreases are not accompanied by significant changes in BMAT volume. These later studies rely on a limited number of subjects but suggest that the initial metabolic status [diabetes (81) or abdominal adipose tissue distribution (82)] can impact on the BMAT amount changes.

In summary, compared with other fat depots, BMAT could, thus, be considered as a strong and reliable indicator of bone integrity that could open new clinical perspectives in the management of osteoporosis (83). Its measurement rather easily performed using MRI should be more systematically analyzed particularly in metabolic diseases considering that most available data have been generated in animal models. In response to metabolic variations, BMAT development differs from other fat depots in both humans and rodents. Yet, it remains conceivable that BMAT expansion may not be completely independent from the WAT distribution. In this connection, BMAT accretion could be triggered when WAT redistribution occurs as in aging. However, the paradoxical situation of anorexia nervosa supports that BMAT formation is dissociated from that of other fat tissues. A mechanistic pathway shared by all physiopathological conditions to explain BMAT development is, thus, still missing.

### BM Adipogenesis

Bone marrow adipogenesis of resident mesenchymal stem cell (MSC) is classically controlled through a transcriptional cascade involving the key transcriptional factors PPARγ and c/EBPα. Within the BM, MSCs reside in the perivascular compartment, at the endosteal surface, and in the marrow space. As in other tissues, BM MSCs represent a heterogeneous cell population with regard to their cell surface marker expression and differentiation potential toward the osteoblast, adipocyte, or chondrocyte lineages (84, 85). It has to be emphasized that a great diversity of adipocyte precursors (distinct MSC populations, pericyte progenitors) for WAT and BAT has been revealed and debated this last decade (3, 44, 86). Interestingly, whereas most adipocytes also develop from adipose tissue-resident progenitors, engrafted BM-derived cells have been shown to generate new adipocytes within WAT in both humans (87) and rodents (88), which argues for another interaction between BM and WAT.

Importantly, adipogenesis is well considered as a competitive process for osteoblastogenesis within the BM. Haplo-insufficiency or overexpression of PPARγ in BM progenitor cells results in increased osteoblastogenesis or adipogenesis, respectively (89). Both *in vitro* and *in vivo* studies have shown that various conditions – such as elevated glucocorticoid levels, estrogen withdrawal, oxidative stress, and immobilization – that promote adipogenesis limit osteoblastogenesis. Conversely, pro-osteogenic factors [growth hormone, insulin-like growth factor 1 (IGF1), Wnt proteins, estrogens, mechanic inputs] decrease adipogenesis (85, 89, 90). Thus, the BM MSC shift toward either lineage results from a complex interplay of systemic and local mediators.

However, the classical view of an unbalanced adipogenesis at the expense of osteoblastogenesis is challenged by other findings. The coexistence within the mouse BM of independent precursor cells committed to each lineage has been shown (91). Both adipocytes and osteoblasts can fully develop from mouse BM MSC in a co-differentiation medium without any alteration of either lineage (92). As described in Section “Physiological BM Cellular Conversion to BMAT,” a part of BMAT development occurs when the bone mass peak is achieved at human puberty and cBMA fully arise without bone loss in rodents. Bone mass can evolve independently of BM adipogenesis in some models (56, 57) or mouse strain (e.g., the C3H/HeJ strain) (93).

In this context, BMAT development cannot represent the only detrimental mechanism. BMA phenotype – which underlies its function – should be considered as a pivotal aspect in bone alterations as emphasized below.

## Specific Properties of BMA Versus Other Adipocytes

### Basic Anatomy of the “BMAT”

Compared with subcutaneous or visceral fat lobules composed of ~80% well-packed adipocytes, BMAs arise loosely connected and scattered among hematopoietic cells. In that respect, such adipocyte distribution may not be referred to as a true “adipose tissue”. Whereas cBMA constitute a rather dense adipocyte area, rBMA are primarily located at the trabecular site where bone remodeling is active, which suggests their involvement in this process. The sympathetic cue is crucial in bone homeostasis (94) but the nerve distribution toward BMAs has not been depicted yet. Compared with hematopoietic areas, BMA-enriched regions are considered to be less vascularized with a lower perfusion as observed in the hip of normal-aged subjects (95). Moreover, vascularization could differ between cBMA and rBMA areas with, surprisingly, a higher capillary density in the cBMA regions in mouse (96).

Bone marrow adipocytes appear filled by a large unilocular lipid vacuole, a typical morphology of white adipocytes. Human BMA diameter can vary from 40 to 65 μm according to the fat infiltration (14), the cellular composition of BM (97), or the analyzed age (14, 98). BMAs are, thus, smaller than subcutaneous and visceral adipocytes in both humans and rodents, with cBMA larger than rBMA (Table 1).

### A White or Brown/Beige Phenotype?

Determining the white or brown/brite phenotype of BMA is crucial with regard to a better understanding of their regulation and function. The development factors (Table 1) and the unilocular morphology of BMAs are more reminiscent of a white adipocyte. However, the preferential distribution of BM adiposity in the appendicular skeleton following a temperature gradient led some authors to propose a thermogenic role for the adipocytes (99, 100). Several gene markers specific for the “brown/beige” lineage were found expressed in the whole tibia BM in young mice. Based on the expression of both white and brown markers upon rosiglitazone treatment, although UCP-1 expression was barely detected, the authors proposed that BMAT has the two phenotypes (101). A high glucose uptake detected within the BM of cold-exposed patients combined with the immuno-detection of UCP-1 protein in the BMA of very young mice convincingly support the potential presence of functional brown/beige BMA in vertebrae (102). However, these results are contradicted by other studies. Several genes of the fatty acid oxidation pathway were found more weakly expressed in isolated BMA from long bones in comparison with isolated visceral adipocytes in mouse (23). Similarly, human BMA isolated from the iliac crest had lower mRNA levels of typical markers of the brown phenotype compared with subcutaneous adipocytes (103). Importantly, a long-term cold exposure of mice led to a marked decline of BMA density in tibiae but a concomitant browning of other WAT depots (29). Altogether, one may suggest that the BMA phenotype varies according to their localization: BMAs that arise in the long bones most likely belong to the white lineage, whereas some BMAs in the vertebrae can exhibit brown-like features. The presence of such thermogenic adipocytes could explain the disappearance of the “yellow BM” in the caudal vertebrae during a temperature rise (104). Indeed, hematopoiesis remains quite efficient in the vertebra BM and brown adipocytes have been proposed to support myelopoiesis through the secretion of some specific adipokines (102).

Most studies characterizing BMA have been performed from long bones or iliac crest samples and have raised some puzzling specificities regarding their paracrine/endocrine and metabolic properties.

### Secretory Profile of BMA

The two main adipokines adiponectin and leptin are detected in isolated mature BMA, could locally promote osteoblastogenesis, and impact on osteoclastogenesis or hematopoiesis (89). However, their mRNA expression levels were found markedly low compared with extramedullary adipocytes in adult healthy donors (103) and normal-aged mice (23).This finding may relate to a lower state of differentiation of BMA as proposed in aging mice (23). Yet BMAT explants from rabbit (distal tibia) or human patients (tibia) were shown to secrete more adiponectin than other WAT depots. Moreover, through the characterization of a mouse model of defective BM adipogenesis, BMAT was revealed as a significant source of serum adiponectin during caloric restriction (34). Even though this contribution to the calorie restriction-induced adiponectinemia rise was not confirmed in rabbits (35), this study underlies a true endocrine status of BMA which can modulate metabolism at the systemic level.

Given the importance of inflammatory factors in the bone loss of post-menopause or aging, the cytokine production by BMA has begun to be assessed. A first antibody-based array study indicates that the cytokine content of BMA diverges from that of subcutaneous adipocytes with a more pro-adipogenic and pro-apoptotic profile in aging mice (105). In another independent transcriptomic study in mice, BMAs were reported to express inflammatory genes, such as TNFα and IL6, at higher levels than visceral adipocytes (23). Surprisingly, in both studies, their expression levels were downregulated during aging, suggesting that this pro-inflammatory profile of BMA is rather early and transient. Of note, another transcriptional analysis failed to detect any further upregulation of these genes in the BMA from normal-aged mice fed with a high-fat diet (24). Interestingly, primary human femoral BMAs express the pro-osteoclastogenic factor RANKL and mediate through a direct cell contact the differentiation of osteoclast precursors (106, 107). RANKL expression was also shown to be associated with BMA differentiation and with Pref1-expressing preadipocytes in the BM of aging mice (108).

**Figure 1:**
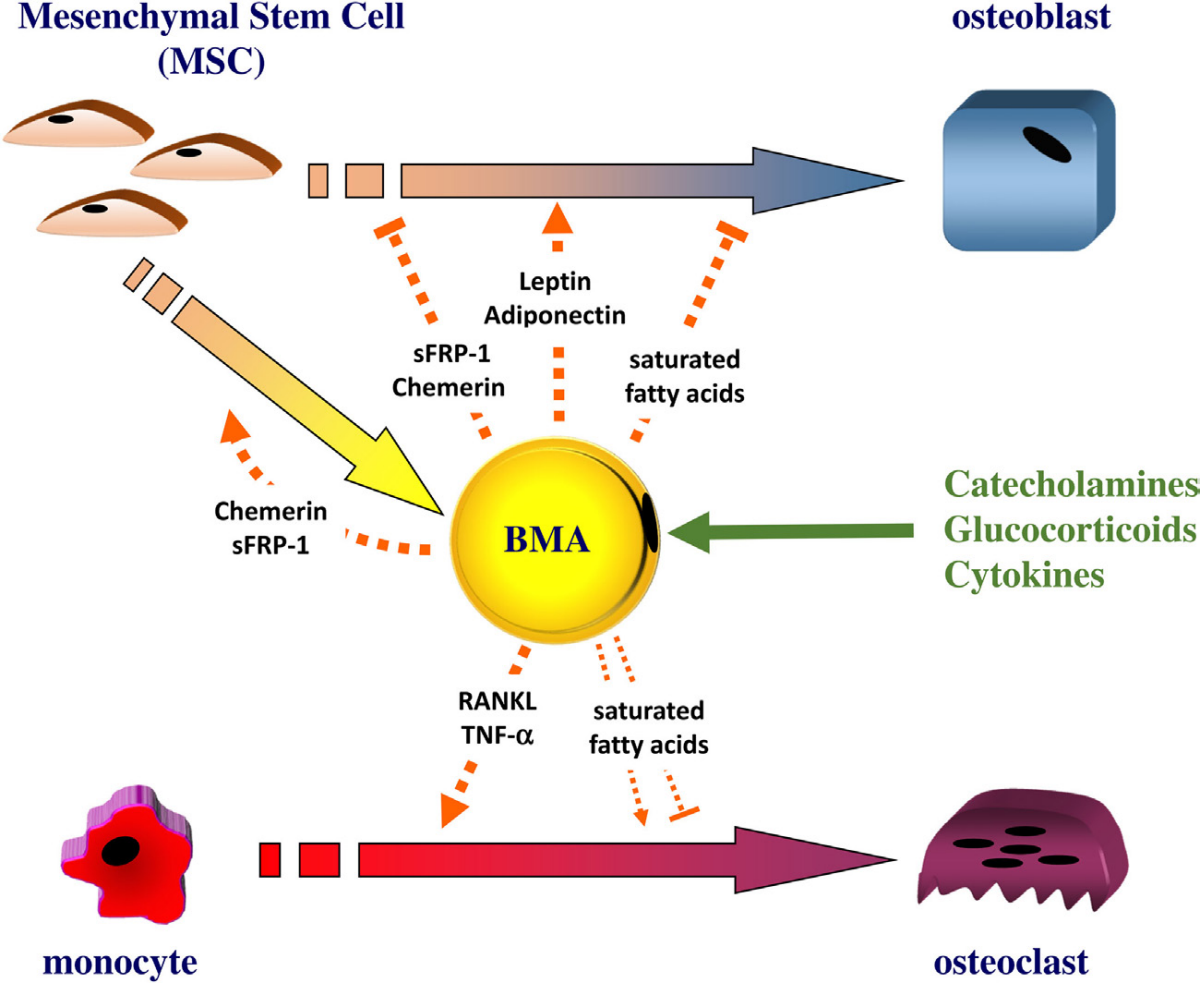
**The bone marrow adipocyte as an active cell in bone remodeling**. The bone marrow adipocyte (BMA) can secrete leptin and adiponectin, which directly stimulate the proliferation and the differentiation of mesenchymal stem cell (MSC) into osteoblast. Other factors, such as the Wnt signaling inhibitor sFRP1 or chemerin, can be released by the BMA: these factors were shown to promote adipogenesis while negatively regulating osteoblastogenesis. As a source of RANKL and TNF-α, the BMA can also support osteoclastogenesis. The BMA appears enriched in saturated fatty acids that can trigger the dysfunction and apoptosis of osteoblasts. Saturated fatty acids could impact on osteoclast differentiation and survival but results are conflicting. The BMA could release these different factors in response to catecholamines, glucocorticoids, and cytokines.

Several *in vitro* studies using notably co-culture models (109, 110) of BM MSC-derived adipocytes and osteoblasts strongly support a deleterious paracrine role of BMA. BM MSC-derived adipocytes have also been reported to secrete factors that alter osteoblastogenesis and favor adipogenesis, such as Wnt signaling inhibitors (111) and chemerin (112) (Figure 1).

### Metabolic Profile of BMA

Adipocytes synthesize and hydrolyze triglycerides in response to insulin and catecholamines, the respective prototypical lipogenic and lipolytic factors. Functional assays to establish the relative capacity of BMA for lipogenesis and lipolysis compared with other fat depots remain scarce due to technical difficulties. Only one early study in rabbits has reported that isolated femoral adipocytes incorporate palmitate into triglycerides at level equivalent to perirenal adipocytes (113). Catecholamine-induced lipolysis appeared lower in femur compared with omental adipose tissue in dogs (114). More indirectly, but in line with their small size, mouse BMAs were shown to express weaker expression of key genes involved in lipogenesis and lipolysis compared with visceral adipocytes (23). Moreover, the metabolic activity of BMA has been indirectly assessed by the adipocyte size and density determination and seems to vary according to their bone localization and the different subtypes in rodents. Indeed, only BMA in the lumbar spine were affected by a chronic administration of a β3-adrenergic agonist (27). In the tibia, cBMA were reported resistant to a long-term cold-induced catecholamine challenge compared with the regulated subpopulation (29). Whether these observations result from the development timing of each BMA subset, a different innervation/vascularization of the BM regions or intrinsic divergences among BMA subtypes has to be further explored. Nevertheless, the relative amount of BMAT compared with other adipose tissues supports that the metabolic influence of BMA is locally confined.

Importantly, the fatty acid composition of BMA is emerging as a key signature of their phenotype. Compared with subcutaneous adipose tissue, lipid extracts of human BM (mainly from proximal tibia or femur) display a higher proportion of saturated fatty acids and a lower amount of monounsaturated ones (115). Based on 1H MRS exploration, the unsaturation index of the vertebral fat fraction is decreased compared with controls and inversely associated with bone loss (51) or fragility fractures (80) in post-menopausal women. Moreover, in healthy adult women, a gradual increase of the unsaturation index is observed from the proximal femur to the most distal part of the tibia, suggesting a differential fatty acid distribution in the BMA subpopulations. Accordingly, this was further examined in several bone sites in rats to reveal a higher unsaturation index in cBMA compared with regulated ones (29). These studies strongly support that BMA associated with bone alterations preferentially store saturated fatty acids. This has a strong pathophysiological relevance since *in vitro* chronic exposure of osteoblasts to saturated fatty acids triggers their dysfunction and apoptosis (116–118). Results are conflicting for the resorbing cells since saturated fatty acids were reported either to reduce osteoclastogenesis (119) or to exert beneficial effects on mature osteoclasts by preventing their apoptosis (120). Though the amount of fatty acids released by BMA appears rather low *in vitro* (121), BMA could contribute to bone alterations through a detrimental fatty acid-mediated process referred as lipotoxicity. Moreover, a specific lipid pattern of BMA may be considered as a discriminative trait, which is rather puzzling considering that dietary fat intake usually impacts on the fatty acid composition of bone (122), adipose tissues, and blood (123). The variation in the BMA fatty acid content could reflect an adaptation to systemic metabolic alterations (79, 80) and/or an intrinsic characteristic through the expression of desaturases (29).

### Regulation of BMA Function

Directly assessing the factors regulating the BMA function as done through *ex vivo* experiments with other fat depots is hampered by the dissemination and the scarcity of BMAT in animal models. Alternatively, indirect measurements of BMA activity through the size and density determination have often been performed using long-term experimentations (27, 29, 40). However, in such studies (27, 40), experiments are often carried out while BM adipogenesis is also initiated, which may interfere with the interpretation of the mature BMA response. One may propose to perform tests when BM adiposity (and mostly rBMA) is fully developed: indeed, a few interventional studies have already uncovered for example a sensitivity to estradiol for BMAT in post-menopausal women (124, 125) or to growth hormone for tibial rBMA in dwarf rats (126). Moreover, using radiolabeled glucose or fatty acid analogs with Positron Emission Tomography imaging could be helpful to decipher the metabolic function and regulation of BMA during acute challenges. So far, information regarding the regulation of BMA function mainly comes from *in vitro* studies (Figure 1).

As mentioned before, like for other WAT, catecholamines or agonists of the β-adrenergic receptors stimulate fatty acid release from BMA both *in vitro* (121, 127, 128) and *in vivo* (114). Surprisingly, insulin addition does not seem necessary for human BM MSC adipogenesis and subsequent lipogenesis *in vitro* (109, 127, 129). However, insulin acutely stimulates glucose uptake (130) and modifies the adipokine pattern (127, 131) *in vitro* and in organotypic cultures supporting that BMAs are sensitive to insulin.

Dexamethasone triggers lipolysis (118) and stimulates the secretion of leptin from human BM MSC-derived adipocytes (118) or human primary cultivated BMAs (131) like reported for extramedullary white adipocytes. Regarding to adiponectin secretion, discrepancies are obtained according to the studied model (118, 131). Dexamethasone also stimulates the expression of RANKL in human primary BMAs (106).

Similarly to white adipocytes, cytokines – including notably TNFα – alter leptin secretion in two cultured models (131, 132), supporting that BMAs are also sensitive to a pro-inflammatory environment.

## Conclusion

Bone marrow adipose tissue is metabolically distinct from other fat depots as indicated by several evidence related to its development and properties. Numerous data from clinical studies, *in vivo*, and vitro models point to an involvement of BMAT in bone remodeling resulting in bone alterations. Besides the participation of the adipogenesis process, BMAs are active cells whose phenotype is strikingly heterogeneous according to the bone site or BM area. Compared with cBMA, rBMA appear highly responsive to diverse stimuli. The first characterizations of BMA as well as their sensitivity to catecholamines and glucocorticoids support that BMA behaves more as a white-like adipocyte whose fatty acid or adipokine release could impact on BM cells (Figure 1). Moreover, its phenotype could also evolve when bone remodeling is altered. Owing to its specific microenvironment, it is expected that osteoblasts, osteoclasts, hematopoietic, and other bone cells reciprocally regulate BMA function. In that respect, a description of BMAT vascularization, innervation, and interaction with BM cells is needed to better comprehend its regulation. Moreover, the relative contribution of BMAT secretions compared with those of extramedullary adipose tissues remains poorly examined, which emphasizes the necessity to deepen the characterization of BMA. So far considering the different physiopathological situations of BMAT development together has not led to a shared hypothesis defining the role of BMA. This actually suggests that, as a true responsive cell, BMA function and regulation could vary according to the pathophysiological context and should be analyzed accordingly.

## Author Contributions

SL drafted the review. TR and SL made the figure. PH, TR, and SL contributed to the design of the review, critically revised it, approved the final version to be published, and agreed to be accountable for all aspects of the work.

## Conflict of Interest Statement

The authors declare that the research was conducted in the absence of any commercial or financial relationships that could be construed as a potential conflict of interest.
